# Congenital hydrocephalus in a trisomy 9p gained child: a case report

**DOI:** 10.1186/s13256-022-03424-5

**Published:** 2022-05-27

**Authors:** Mikkel Bak Henningsen, Helga Angela Gulisano, Carsten Reidies Bjarkam

**Affiliations:** Department of Neurosurgery, Aalborg University Hospital, Hobrovej 18-22, 9000 Aalborg, Denmark

**Keywords:** Case report, Choroid plexus hyperplasia, Congenital heart disease, Endoscopy, Hydrocephalus, Trisomy 9p, VA shunt

## Abstract

**Background:**

Hydrocephalus caused by excessive liquor production due to choroid plexus hyperplasia is a rare condition that may necessitate unusual treatment paradigms. It can be seen in trisomy 9p where coexisting congenital heart disease additionally may complicate the therapeutic approach as illustrated in the current case report.

**Case presentation:**

At 20 months of age, a Caucasian girl with trisomy 9 and family history of an older brother and twin sister having the same syndrome displayed sign of congenital hydrocephalus due to increasing head circumference. Magnetic resonance imaging revealed enlarged lateral ventricles and a prominent choroid plexus, and the girl was treated with a ventriculoperitoneal shunt, which 2 days later had to be replaced with a ventriculoatrial shunt as cerebrospinal fluid formation greatly exceeded the ability of the patient’s abdominal absorptive capability. At 16 years of age, the patient was diagnosed with cardiomyopathy and diminished ejection fraction. Some months later, she was admitted to the neurosurgical ward showing signs of shunt dysfunction due to a colloid cyst in the third ventricle. Cystic drainage through endoscopic puncture only helped temporarily. Revision of the shunt system showed occlusion of the ventricular drain, and replacement was merely temporary alleviating. Intracerebral pressure was significantly increased at around 30 mmHg, prompting externalization of the drain, and measurements revealed high cerebrospinal fluid production of 60–100 ml liquor per hour. Thus, endoscopic choroid plexus coagulation was performed bilaterally leading to an immediate decrease of daily cerebrospinal fluid formation to 20–30 ml liquor per hour, and these values where stabilized by pharmaceutical treatment with acetazolamide 100 mg/kg/day and furosemide 1 mg/kg/day. Subsequently a ventriculoperitoneal shunt was placed. Follow-up after 1 and 2 months displayed no signs of hydrocephalus or ascites.

**Conclusions:**

High cerebrospinal fluid volume load and coexisting heart disease in children with trisomy 9p may call for endoscopic choroid plexus coagulation and pharmacological therapy to diminish the daily cerebrospinal fluid production to volumes that allow proper ventriculoperitoneal shunting.

## Introduction

In children with trisomy 9p, congenital hydrocephalus due to choroid plexus hyperplasia is well documented alongside a variety of phenotypical manifestations including craniofacial dysmorphic features, growth retardation, mental retardation, hand and foot anomalies [1, 3, 8, 9, 16], and congenital heart diseases including ventricular septal defect (VSD), atrial septal defect (ASD), hypoplastic left heart, and cardiomyopathy [3, 8, 9, 13, 14, 16].

Hydrocephalus is commonly treated by implantation of a ventriculoperitoneal (VP) shunt). In patients with choroid plexus hyperplasia, however, the CSF production can exceed several liters a day, ultimately causing accumulation of CSF in the abdominal cavity and ascites [1, 4–7]. Optional but rarely used treatment options such as ventriculoatrial (VA) shunt, choroid plexus coagulation, and pharmacological treatment may thus be needed to control choroid plexus hyperplasia-derived hydrocephalus in trisomy 9p [1, 4–15, 15, 16]. However, the known risk of VA shunt-associated cardiovascular complications such as endocarditis, heart failure, intraatrial thrombus, and pulmonary hypertension may exclude this treatment modality in trisomy 9p patients with cooccurring congenital heart disease [4, 5, 8, 9, 13, 14, 16], as illustrated by the current case report.

## Case presentation

We report the case of a Caucasian girl with trisomy 9p and family history of an older brother and twin sister having the same syndrome [1]. The patient was born as twin A (birth weight 1800 g, length 45 cm) and showed dysmorphic features from birth including a large fontanel, low-hanging ears, and a bulbous nose (cf. Fig. 2C in Ref. [1]). Clinodactyly, for example, short fifth fingers with only on flexion joint and palms with singular crease, was likewise noted [1]. At birth, the head circumference was below average and like twin B.

**Fig. 1:**
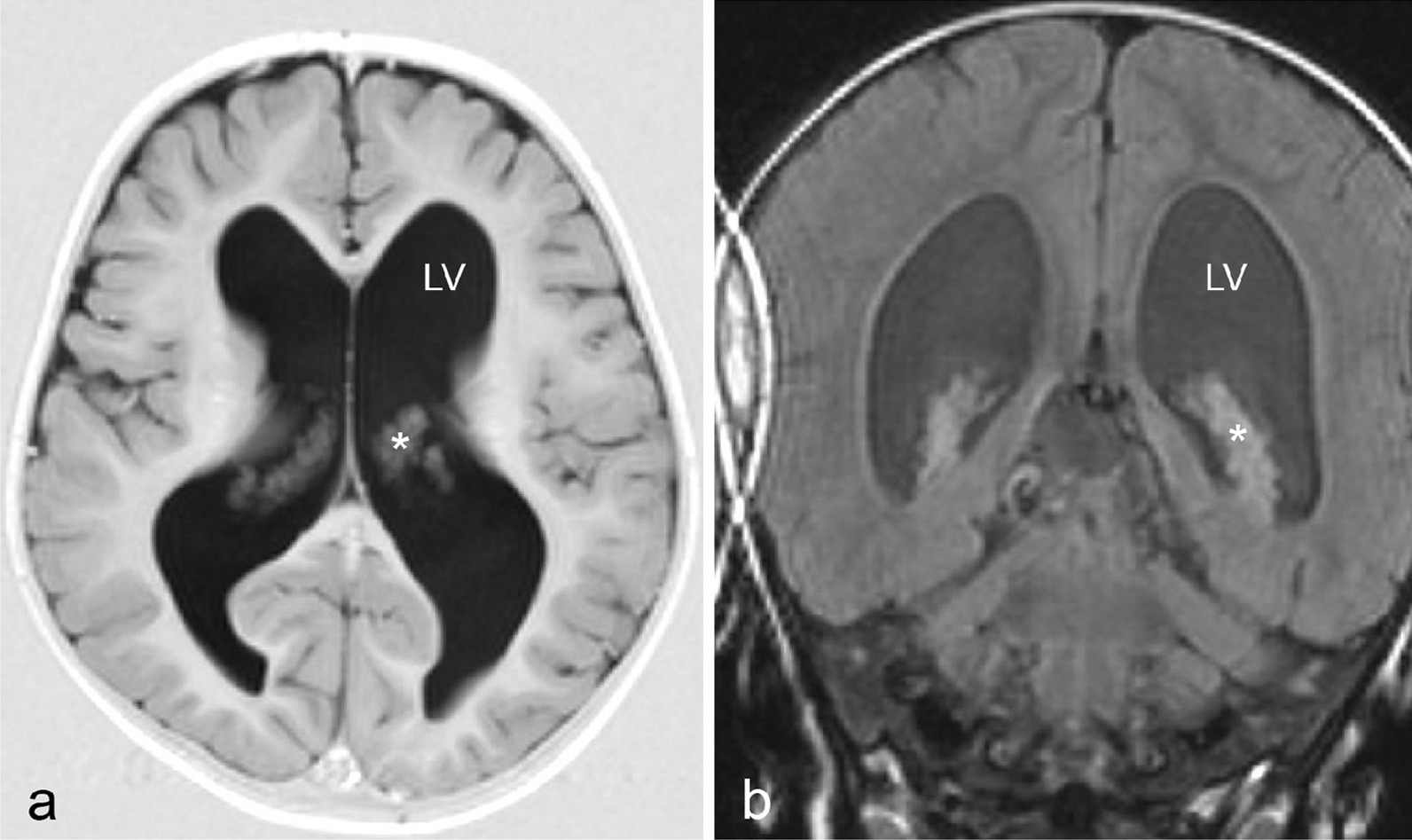
T1-weighted axial (**a**) and coronal (**b**) MRI images obtained at patient age 2 years, showing hydrocephalus due to enlarged lateral ventricles (LV) encompassing a prominent choroid plexus (*)

At 20 months, her general practitioner discovered an acceleration in head circumference growth to two SD above average, compared with her twin sister (average for age). It was further noted that the girl was not able to walk independently yet, but apart from this there was no signs of neurological compromise, pain, or growth retardation. MRI of the brain showed enlarged lateral ventricles with a prominent choroid plexus (Fig. 1). At first, aqueduct stenosis was suspected, but this was later disproved by ventriculoscopy and ventriculography. Therefore, the patient was treated with a VP shunt at 2 years of age. On the second postoperative day, she showed signs of massive CSF accumulation into the abdominal cavity and fluid leakage through the abdominal cicatrices. Revision of the shunt showed no leakage and confirmed a functioning shunt system. The clinicians concluded that the CSF formation greatly exceeded the ability of the patient’s abdominal absorptive capability. Subsequently, the VP shunt was replaced with a VA shunt during the same admission.

At 6 years of age, the girl was now attending special classes due to slight psychomotor compromise, but she had language and independent ambulatory function. However, due to irregular episodes of headache and vomiting, hydrocephalus was suspected, resulting in an X-ray image showing displacement of the atrial part of the drain due to growth. Surgically prolongation of the drain was successfully performed, relieving the pressure symptoms.

At 14 years of age, irregular episodes of headache and vomiting reappeared. Shunt dysfunction was suspected, and cerebral MRI showed a large occipital cyst in contact with the right lateral ventricle. Operative fenestration was performed, successfully relieving the symptoms.

At 16 years of age, the patient was diagnosed with cardiomyopathy based on transthoracic echocardiography (TTE) showing dilation of the left ventricle and diminished ejection fraction (EF, 49%). The TTE was performed because her twin sister had been diagnosed with cardiomyopathy several years before. Subsequent pharmacological treatment with ramipril was started.

**Fig. 2:**
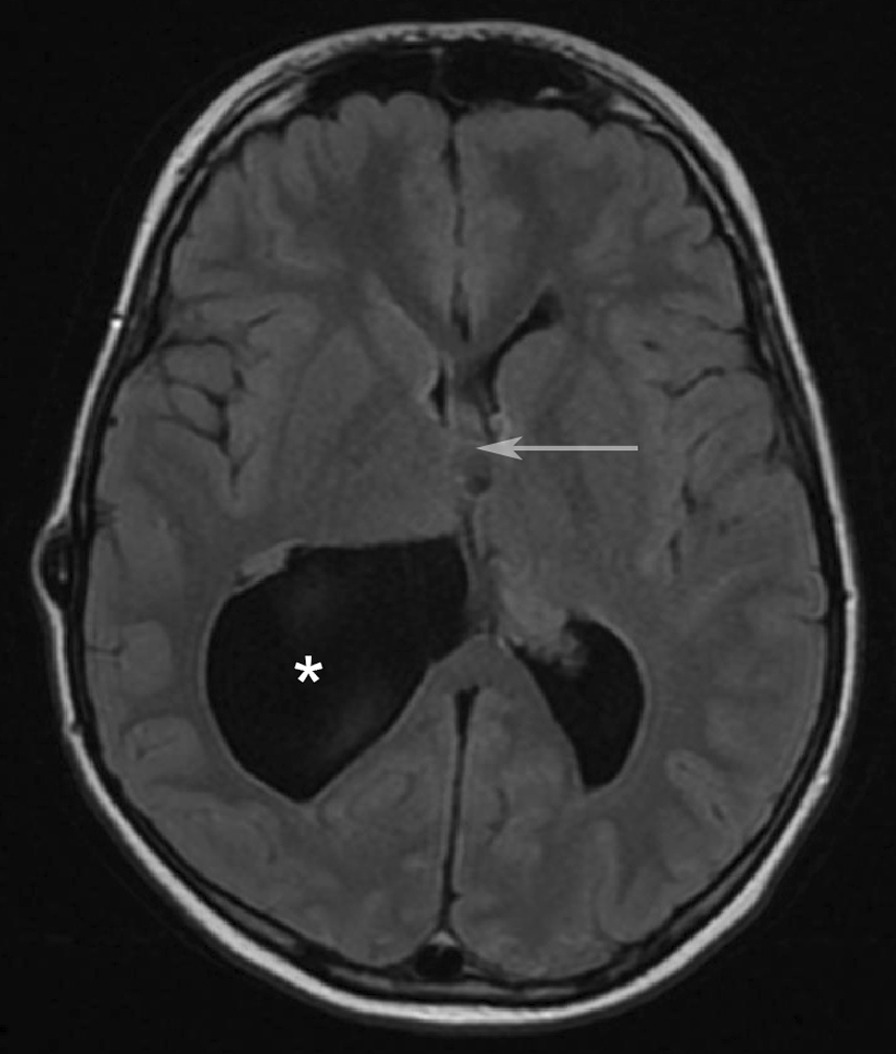
T1-weighted axial MRI obtained at age 16 years, showing occlusion of the foramen of Monro by a colloid cyst (arrow) and a cystic enlargement of the posterior horn of the right lateral ventricle. * = lateral ventricle

Some months later, the patient was admitted to the neurosurgical ward showing signs of shunt dysfunction (that is, headache, vomiting, and tiredness). The neurological examination did not reveal any specific compromise, but subsequent ophthalmological examination (fundus photography) revealed papilledema, supporting the clinical suspicion of raised intracranial pressure (ICP). MRI of the brain was performed and showed a colloid cyst in the third ventricle, possibly occluding the foramen of Monro (Fig. 2).

Cystic drainage through endoscopic puncture only helped temporarily. Revision of the shunt system showed occlusion of the ventricular drain, and replacement was likewise only temporary alleviating as the symptoms of headache, vomiting, and tiredness reappeared within 24 hours. Accordingly, the ICP was monitored by placement of an intracranial ICP monitor and found to be significantly increased at around 30 mmHg, prompting externalization of the drain and measurements of CSF production. Externalization of the drain relieved all clinical symptoms, but measurements revealed high CSF production of 60–100 ml liquor per hour. Endoscopic visualization showed that the choroid plexus was enlarged, hypertrophic, and well vascularized. Choroid plexus coagulation was performed bilaterally in the lateral and temporal horn of the ventricles, leading to an immediate decrease of daily CSF formation to 20–30 ml liquor per hour. Pharmaceutical treatment with acetazolamide 100 mg/kg/day and furosemide 1 mg/kg/day [12] diminished daily CSF production to approximately 300 ml. Subsequently a VP shunt (DeltaSnap 1.0) was placed, and the remaining admission was uneventful.

Follow-up after 1 and 2 months displayed no signs of hydrocephalus or ascites. No complications or neurological deficits were found. At a regular follow-up 10 months later, it was noted that the girl trived and was more active and less tired in school and at home.

## Discussion and conclusions

Hydrocephalus can be considered as a condition caused by disordered CSF homeostasis that results in expansion of the cerebral ventricles [2, 4–7]. It may accordingly arise from an increase in CSF secretion and/or an obstruction of flow in the ventricular–subarachnoid pathway, and/or a decrease in drainage to the venous system [2]. However, although the choroid plexus with a secretory rate of 0.4 ml minute^−1^ g tissue^−1^ is among the most efficient secretory tissues in our body and only paralleled by the cells of the renal proximal tubule and pancreatic ducts, one rarely encounters hydrocephalus caused by CSF hypersecretion [2, 4, 5]. CSF hypersecretion can be caused by secreting choroid plexus neoplasms (papilloma or carcinoma), which account for 0.4–0.6% of all intracranial neoplasms, or by choroid plexus hyperplasia, where the number of normal productive epithelial cells is increased [4]. To date, hydrocephalus caused by choroid plexus hyperplasia has been described in fewer than 30 patients [4–7, 10]. Ten of these patients have been cytogenetically characterized, revealing five with trisomy 9p and four with tetrasomy 9p [1, 4, 7, 10]. Similarly gain of 9p has been demonstrated in 45% of hypersecretive choroid plexus tumors [12], indicating that genetic material on the short arm of chromosome 9 may be important for choroid plexus growth and secretion [1, 12]. However, note that, among the 200 known reported cases of 9p tri- or tetrasomy, only 10 cases of choroid plexus hyperplasia have been described [1, 4–7, 10], whereas cases with corpus callosum anomalies and Dandy–Walker malformation causing obstructive hydrocephalus are seen more commonly [1].

Treatment of congenital hydrocephalus due to choroid plexus hyperplasia in patients with tri- and tetrasomy 9p is complicated by the resulting CSF volume load, which may reach a daily volume of between 2000 and 5000 ml [1, 4–7, 10]. Such volumes can rarely be absorbed by the peritoneal cavity, and traditional VP shunting in these patients will therefore result in abdominal ascites necessitating either choroid plexus plexectomy/coagulation and/or placement of a VA shunt [1, 4–7, 10]. Plexectomy or endoscopic plexus coagulation can be curative, although it is stated in the case review by Hallaert *et al.* (2012) that, among 16 treated patients, 11 remained in need of a shunt after the procedure [5].

The recent case report by Kasper *et al.* (2020) confirms the difficulties in the treatment of trisomy 9p associated with CSF hypersecretion, and the authors advocate placement of a VA shunt in this condition [7]. It is, however, estimated that 40–60% of the 9p tri- and tetrasomy cohort have coexisting heart disease [1, 3, 8, 9, 13, 15]. Placement of a VA shunt in these patients, as secondarily performed in our case [1], may accordingly be less optimal, necessitating VA shunt removal and subsequent choroid plexus coagulation and/or pharmacological therapy with acetazolamide and furosemide to diminish daily CSF production to volumes that allow proper VP shunting [4–7, 11, 15].

## Data Availability

Access to further information can be obtained by contacting the corresponding author.

## Abbreviations

CSF: Cerebrospinal fluid
EF: Ejection fraction
ICP: Intracranial pressure
LV: Lateral ventricle
MRI: Magnetic resonance imaging
SD: Standard deviation
TTE: Transthoracic echocardiography
VA: Ventriculoatrial
VP: Ventriculoperitoneal
